# How neurons adjust to diurnality

**DOI:** 10.7554/eLife.74704

**Published:** 2021-11-30

**Authors:** Gabriele Andreatta, Charles N Allen

**Affiliations:** 1 Department of Microbiology, Immunobiology and Genetics, Max Perutz Labs, University of Vienna Vienna Austria; 2 Oregon Institute of Occupational Health Sciences, Oregon Health & Science University, Department of Behavioral Neuroscience Portland United States

**Keywords:** *R. pumilio*, diurnality, circadian rhythms, suprachiasmatic nucleus, electrical activity, mathematical modelling, Other

## Abstract

Being active during the day requires a slow-closing ion channel that dampens the activity of neurons in a specific area of the brain.

**Related research article** 2021. Bano-Otalora B, Moye MJ, Brown TM, Lucas RJ, Diekman CO, Belle MDC. 2021. Daily electrical activity in the master circadian clock of a diurnal mammal. *eLife* **10**:e68179. doi: 10.7554/eLife.68179

Most humans are diurnal, meaning they are usually awake during the day and asleep at night. However, this is not the case for many other animals, which enjoy nightlife and rest throughout the day. So how do brains determine whether we are nocturnal or diurnal?

Many physiological processes, such as wakefulness or sleep**,** are synced to the hours of daylight and darkness. These activities are regulated by molecular oscillators called circadian clocks, which consist of positive and negative feedback loops of gene transcription and protein translation that allow processes to take place with a ~24 hour periodicity. Like instruments in an orchestra, the concert of ‘ticks’ generated by these clocks, which are found throughout the body, must be harmonized to coordinate the activities of different organs.

In mammals, the conductor of this symphony is the ‘master circadian clock’ which resides in the suprachiasmatic nucleus (SCN), a cluster of around 20,000 neurons in a region of the brain called the hypothalamus. Each neuron in the SCN times its electrical activity to the day-to-night cycle, ultimately generating rhythmic inputs that the body obeys (Reppert and Weaver, 2002).

Most of the current knowledge about these neurons and how they synchronise derives from studies on nocturnal rodents, like mice, rats, and hamsters (Carmona-Alcocer et al., 2020). In diurnal species, only recordings from the entire SCN are available. Surprisingly, such data suggest that the master clock neurons in diurnal and nocturnal animals have similar molecular oscillators and electrical properties (Sato and Kawamura, 1984; Yan et al., 2020).

However, information on single neurons is lost in these recordings, limiting our understanding of the cell-specific mechanisms that potentially determine whether an animal is nocturnal or diurnal. Now, in eLife, a group of scientists led by Robert Lucas, Casey Diekman, and Mino Belle – including Beatriz Bano-Otalora and Matthew Moye as joint first authors – report on the electrical properties of individual SCN neurons in the diurnal four-striped mouse *Rhabdomys pumilio* (Bano-Otalora et al., 2021).

First, the team (who are based at the Universities of Manchester and Exeter, the New Jersey Institute of Technology and Merck) recorded the electrical activity of single neurons using a technique called whole-cell patch clamping. This revealed that, like in nocturnal animals, single neurons in the SCN of diurnal animals were more excitable and active during the day than at night (Allen et al., 2017; Figure 1).

This suggests that both diurnal and nocturnal species use similar mechanisms to generate daily rhythms in the brain, irrespectively of when the animals are active. Moreover, the SCN neurons of *R. pumilio* reacted to excitatory electrical stimuli – stimuli that dissipate the membrane potential (which at rest is about –70 mV) and trigger an action potential that relays the signal to downstream neurons – similarly to the SCN neurons of nocturnal rodents (Belle et al., 2009).

**Figure 1.**
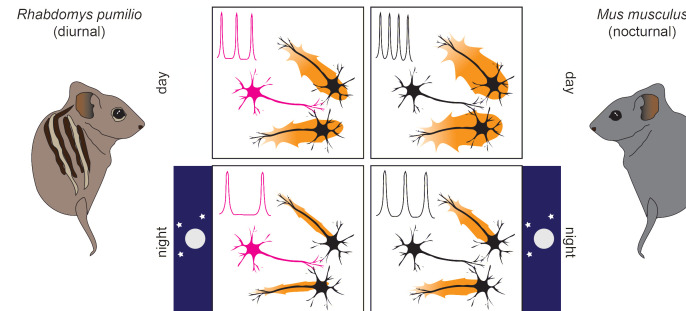
Neurons with unique properties regulate suprachiasmatic nucleus (SCN) excitability to accommodate a diurnal lifestyle. In both diurnal (left) and nocturnal (right) mammals, SCN activity (orange halos around neurons) is higher during the day (top, larger halos) than at night (bottom, smaller halos). In the *R. pumilio* SCN (left side) – but not in the mouse SCN (right side) – one out of three neurons (in magenta) shows a prolonged resting phase after inhibitory stimuli. This resting phase delays action potential firing, the frequency of which is shown in a simplified scheme on the upper left corner of each panel. This ‘brake’ to SCN excitability operates irrespective of the time of day, leading to reduced activity in the SCN of *R. pumilio* overall (left panels, smaller halos) while maintaining distinct excitation patterns between day and night. This activity pattern is likely an adaptation for diurnality.

However, Bano-Otalora et al. found that some neurons displayed a peculiar electrical behaviour that had not been observed in the SCN of nocturnal animals. An inhibitory stimulus is one that makes the membrane potential of a neuron more negative, making an action potential harder to trigger, and reducing the activity of downstream neurons. When this type of stimulus was applied to the SCN of the diurnal *R. pumilio*, one third of the neurons exhibited a prolonged ‘rest’ during which they could not become excited, thus delaying new action potentials (Figure 1).

Interestingly, these neurons did not change during the day, indicating that their behaviour was not the result of day-to-night changes in the environment, but rather a specific feature of that neuron. Bano-Otalora et al. suggest the presence of such neurons might be critical for mammals to live in daylight: during the day, these neurons can become desensitized to the prolonged high levels of light (Yan et al., 2020), while at night, the effect of inhibitory signals is magnified so that activating the SCN requires a more powerful stimulus.

But how are these neurons delaying their firing? To overcome the challenges of directly studying the complex kinetic properties of the various ion channels that regulate the activity of neurons, Bano-Otalora et al. created a computer model of the *R. pumilio* SCN neurons. This algorithm was designed using the information obtained in the previous experiments that described the main electrical properties of SCN neurons. Running it under different conditions allowed Bano-Otalora et al. to determine what properties SCN neurons need to exhibit to match the experimental results. With this approach, the activity of a specific potassium channel was identified that potentially explains the delay in neuronal activity observed in certain SCN neurons of *R. pumilio*.

These potassium channels close slowly, and the current generated while they close could prolong the refractory period (the time it takes before the neuron is ready to be excited again), preventing a fast re-firing. Interestingly, the activity of this channel was known to delay neuronal firing in other areas of the brain of nocturnal animals (Burdakov et al., 2004; Tarfa et al., 2017), but not in the SCN, suggesting that this is a unique property of diurnal animals. To experimentally confirm the prediction of the SCN model, Bano-Otalora et al. treated slices of *R. pumilio* SCN with a drug that inhibits this potassium channel. This resulted in the ‘lazy’ neurons behaving like the rest of the neurons in the SCN, with no delay in firing.

These results indicate that the neurons in the SCN with the peculiar electrical current provide a steady ‘brake’ to SCN excitability, potentially allowing these cells to become desensitized to daylight and decreasing the likelihood they will activate other neurons during the night (Figure 1). Taken together, the findings of Bano-Otalora et al. provide the first mechanistic insights into the neuronal adjustments present in the brains of diurnal animals. In this context, tuning down neuronal excitability within the master circadian clock seems key to accommodating a diurnal lifestyle. However, it remains unclear which mechanisms switch wakefulness and sleep between days and nights, respectively, allowing animals to take advantage of this newly discovered brain feature.
